# Association of pre-pregnancy body mass index, gestational weight gain with cesarean section in term deliveries of China

**DOI:** 10.1038/srep37168

**Published:** 2016-11-22

**Authors:** Chao Xiong, Aifen Zhou, Zhongqiang Cao, Yaqi Zhang, Lin Qiu, Cong Yao, Youjie Wang, Bin Zhang

**Affiliations:** 1 Department of Maternal and Child Health, School of Public Health, Tongji Medical College, Huazhong University of Science & Technology, Wuhan, China; 2 Wuhan Medical & Healthcare Center for Women and Children, Wuhan, China.

## Abstract

China has one of the highest rates of cesarean sections in the world. However, limited epidemiological studies have evaluated the risk factors for cesarean section among Chinese women. Thus, the aim of this cohort study was to investigate the associations between pre-pregnancy BMI, gestational weight gain (GWG) and the risk of cesarean section in China. A total of 57,891 women with singleton, live-born, term pregnancies were included in this analysis. We found that women who were overweight or obese before pregnancy had an elevated risk of cesarean section. Women with a total GWG above the Institute of Medicine (IOM) recommendations had an adjusted OR for cesarean section of 1.45 (95% CI, 1.40–1.51) compared with women who had GWG within the IOM recommendations. Women with excessive BMI gain during pregnancy also had an increased risk of cesarean section. When stratified by maternal pre-pregnancy BMI, there was a significant association between excessive GWG and increased odds of cesarean section across all pre-pregnancy BMI categories. These results suggest that weight control efforts before and during pregnancy may help to reduce the rate of cesarean sections.

During recent decades, the rate of cesarean sections has risen markedly in both developed and developing countries, especially in China. Among women in urban areas of China, the rate of cesarean sections rose from 14.9% to 54.1% between 1993 and 2008. According to a survey conducted by the World Health Organization (WHO), nearly half of all the infants born in China were delivered by cesarean section during 2007–2008, which was three times higher than the WHO’s recommended cesarean rate of 15%^1^. At present, China has one of the highest rates of cesarean sections in the world. Previous studies have indicated that cesarean sections performed without medical indication may be associated with increased risk of maternal complications such as infection, haemorrhage, or even death^2^. The performance of cesarean sections may also be associated with an increased healthcare system burden, and women who have undergone cesarean section may be at increased risk of abnormal placentation during subsequent pregnancies^3^,^4^. Additionally, infants delivered by cesarean section may be more likely to develop respiratory problems, obesity, and other metabolic syndromes later in life^5^. Therefore, identifying the risk factors for cesarean section, especially those that are modifiable is important for the formulation of interventions to reduce the rate of cesarean sections.

Previous studies have identified an association between maternal overweight/obesity and risk of cesarean section^6^; however, most of these studies were conducted in developed countries, and women of reproductive age in developing countries generally have a lower body mass index (BMI) than those in developed countries^7^. As maternal weight during pregnancy is influenced by a combination of both pre-pregnancy weight and gestational weight gain (GWG), there have been increasing concerns regarding whether GWG may influence cesarean section risk and, therefore, whether controlling GWG can reduce the risk of undergoing cesarean delivery. However, previous studies have mainly focused on GWG in women who were overweight or obese, and few studies have investigated the risk of cesarean section among women of normal weight or underweight status who gained excessive weight during pregnancy^8^. Therefore, the independent and joint associations between pre-pregnancy BMI, GWG and cesarean section deliveries have not been well studied, especially in developing countries.

In this cohort study, we sought to investigate the potential associations between pre-pregnancy BMI, GWG, gestational BMI gain and risk of cesarean delivery in women with term pregnancies in China.

## Results

Table 1 shows the distribution of selected characteristics among women included in the cohort. Of the 57,891 included women, 34,177 underwent cesarean section. Women aged over 30 years, who delivered an infant that was male or over 4000 g birth weight, and who were overweight/obese before pregnancy were more likely to undergo cesarean section. The mean total GWG among women underwent cesarean section was 18.00 ± 7.24 kg, which was higher than the mean GWG among those who delivered vaginally (16.27 ± 6.64 kg).

Table 2 presents the associations between pre-pregnancy BMI, GWG, and gestational BMI gain and the risk of cesarean section. In the model adjusted for potential confounders including maternal age at delivery, education level, parity, offspring gender and birth weight, pre-pregnancy BMI, GWG and gestational BMI gain were all positively associated with the risk of cesarean section. Significantly increased odds of cesarean section were observed among women who were overweight or obese prior to pregnancy (adjusted ORs for overweight and obese women were 1.73 and 2.90, respectively) when compared with women who had a normal pre-pregnancy BMI. The adjusted OR for cesarean section among women who had GWG above the IOM recommendations was 1.45 (95% CI, 1.40–1.51) compared with women who had GWG within the recommendations. Women with a BMI gain greater than 8 kg/m^2^ during pregnancy had an adjusted OR for cesarean section of 2.01 (95% CI, 1.90–2.12) compared with women who had a BMI gain less than 4 kg/m^2^. The odds of cesarean section increased as gestational BMI gain increased (*P* value for trend < 0.01).

The results of the analysis of the associations between GWG and gestational BMI gain and the risk of cesarean section stratified by pre-pregnancy BMI are presented in Table 3. Women with higher GWG and BMI gain had significantly increased odds of undergoing cesarean section across all pre-pregnancy BMI statuses. However, the odds ratio for the association between higher BMI gain and cesarean section delivery decreased as pre-pregnancy BMI increased. In particular, women who were underweight before pregnancy and had excessive BMI gain during pregnancy (≥8 kg/m^2^) had the highest odds of cesarean delivery (adjusted OR, 2.49, 95% CI, 2.07–3.01), whereas the corresponding ORs were still elevated but not as strong among women with excessive BMI gain during pregnancy who were overweight or obese before pregnancy (*P* value for heterogeneity < 0.01).

## Discussion

In this cohort study conducted among Chinese women, we found that maternal overweight/obesity prior to pregnancy was independently associated with increased odds of cesarean section. This result was consistent with those of previous studies^9^,^10^. Biologically, maternal overweight/obesity has been found to be associated with several pregnancy complications and adverse birth outcomes that may greatly increase the likelihood of cesarean section, such as hypertensive disorders of pregnancy and macrosomia^11^,^12^. However, in our study, despite excluding women with pregnancy complications, such as hypertensive disorders of pregnancy, and adjusting for birth weight, the odds of cesarean section remained higher in overweight/obese women, which was consistent with those of previous studies^13^. Compared to normal weight women, overweight/obese women are clinically indicated for cesarean delivery due to complications during vaginal delivery and longer first stage of labour observed in this population^14^. Additionally, previous studies have shown that women who were overweight or obese were more likely to have anxiety or depressive symptoms during pregnancy^15^,^16^, which may also play a role in their elevated risk for cesarean section.

Prior studies as well as this study have consistently shown that being overweight/obese prior to pregnancy was associated with elevated risk of cesarean section. This finding can provide a potential mode of intervention to reduce rates of cesarean sections. However, as there has been limited pre-pregnancy weight loss interventions for women of reproductive age, increasing focus has been directed towards determining whether gestational weight gain (GWG) may influence the likelihood of cesarean section and to what extent controlling GWG can reduce this likelihood. Studies conducted in developed countries have shown that GWG was an independent risk factor for cesarean section^6^,^17^, suggesting that as weight gain increased, the predicted probability of undergoing cesarean section also increased. The strong association observed between increasing GWG and increasing odds of cesarean section in our study was in accordance with the findings of previous studies. Our study indicated that excessive total GWG was independently associated with significantly increased odds of cesarean section. As BMI has been considered by some studies to be a better indicator of body fat than weight alone^8^, we also classified pregnancy weight gain according to net change in BMI, and similarly, excessive BMI gain during pregnancy was shown to be associated with elevated odds of cesarean section. Excessive weight gain during pregnancy might increase the risk of several adverse outcomes such as fetal distress, low birth Apgar score and large for gestational age births^18^,^19^, which may result in cesarean delivery. Furthermore, as excessive GWG may be associated with increased adipose tissue and cholesterol deposits in the myometrium, which may in turn inhibit the myometrial contractility, thereby potentially impeding the labour process, complicating vaginal delivery^14^,^20^, and increasing the need for cesarean delivery. Weight control may be much more feasibly achieved during pregnancy than before pregnancy because while some women may not seek pre-pregnancy care, almost all women receive several antenatal care services.

Some evidence has suggested that pre-pregnancy BMI may modify the association between gestational weight gain and mode of delivery^17^. To explore this possibility, we analysed the association between weight gain and cesarean section stratified by maternal pre-pregnancy BMI status and found a significant association between excessive GWG during pregnancy and elevated odds of cesarean section across all pre-pregnancy BMI statuses. When comparing the adjusted odds ratios, women who were underweight before pregnancy with excessive BMI/weight gain during pregnancy had greater odds of cesarean delivery than did overweight/obese woman with excessive BMI gain during pregnancy. This result is consistent with the findings of a previous study conducted in the USA, which indicated that women with normal pre-pregnancy BMI and excessive weight gain during pregnancy had a greater risk of cesarean section compared with their obese counterparts^8^. This result suggests that weight control counselling is not only necessary for obese or overweight women but for women of any pre-pregnancy BMI status, as significantly greater odds of cesarean delivery were consistently identified in association with excessive GWG regardless of pre-pregnancy BMI status.

This was a cohort study with a relatively large sample size, which allowed us to control for some potential confounding factors that may be associated with mode of delivery. However, some limitations should be noted when interpreting the results of our study. Firstly, pre-pregnancy weight was assessed based on subject recall, which may have resulted in recall bias. However, given that the pre-pregnancy weight data were collected during the first trimester (before birth outcomes were known), the potential misclassification was likely to be non-differential. Secondly, we were not able to distinguish between elective and emergency cesarean deliveries in our study due to a lack of relevant data, and further prospective research in this area is recommended.

In conclusion, we conducted a population-based cohort study in China to explore the associations between pre-pregnancy BMI and GWG and the odds of cesarean section. We found that pre-pregnancy BMI, GWG, and gestational BMI gain were all independently associated with increased odds of cesarean section. Our results indicate that maternal overweight/obesity, GWG, and gestational BMI gain should be considered in concert to reduce the likelihood of cesarean section. Weight restrictions before and during pregnancy are both important in reducing the rate of cesarean sections; promoting weight and BMI control may be particularly important during pregnancy as it is much more feasible during pregnancy than pre-pregnancy. The findings of our study suggest that weight control during pregnancy should be included in routine prenatal care.

## Methods

### Study Population

This was a cohort study conducted in Wuhan, China. We used the data from the Maternal and Children Healthcare Information Tracking System of Wuhan, which includes information on maternal demographic characteristics, medical history, prenatal examinations and delivery information from all 93 hospitals and 121 community health centres in Wuhan. Women who lived in the urban areas of Wuhan during pregnancy and who delivered a singleton infant at 37–42 gestational weeks between January 2012 and June 2013 were eligible for inclusion in our study. Women with chronic diseases (i.e., diabetes, hypertension and heart disease) identified prior to or during pregnancy and who gave birth to a stillborn infant or an infant with birth defects were excluded. Additionally, participants with missing anthropometric data (i.e., maternal height, pre-pregnancy weight and weight at delivery) were excluded.

A total of 57,891 women met the eligibility criteria and were included in this study. The research protocol was approved by the Medical Ethics Committee of the School of Public Health, Tongji Medical College, and all methods applied in this study were carried out in accordance with the approved guidelines. Informed consent was obtained from all subjects before participation.

### Assessment of study variables

Information on delivery mode was obtained from birth records. Both primary and repeat cesarean sections were included in the outcome measure. The birth weight and gender of the infants were obtained from birth records. Gestational age was calculated based on the delivery date and the date of the last recorded normal menstrual period. Pre-pregnancy weight and height were self-reported during the first antenatal care visit (usually during the first trimester). Pre-pregnancy BMI was calculated as weight (kg)/height (m^2^) and then categorized into four groups based on the recommendations of the Working Group on Obesity in China: underweight (<18.5 kg/m^2^), normal weight (18.5–23.9 kg/m^2^), overweight (24–27.9 kg/m^2^), or obese (≥28 kg/m^2^)^21^.

Maternal weight at delivery was measured within 3 days before the delivery date. GWG was calculated by subtracting the pre-pregnancy weight from the maternal weight at delivery and then categorized according to the recommendations of the Institute of Medicine (IOM) (2009)^22^. GWG within the IOM recommendations was defined as gains of 12.5–18 kg, 11.5–16 kg, 7–11.5 kg, and 5–9 kg for underweight, normal weight, overweight, and obese women, respectively.

Gestational BMI gain was categorized as class I (<4.0 kg/m^2^), class II (4.0–5.9 kg/m^2^), class III (6.0–7.9 kg/m^2^), or class IV (≥ 8 kg/m^2^) based on the results of our previous study^23^. Each one point increase in BMI was roughly equivalent to a 2.5 kg gain in weight, as determined using the Chinese national average weight and height for females of reproductive age (158 cm, 54 kg)^21^.

### Statistical Analysis

Unconditional logistic regression analyses were conducted to calculate odds ratios (ORs) and 95% confidence intervals (CIs) for the associations between cesarean section and maternal pre-pregnancy BMI, GWG and BMI gain during pregnancy. The regression models were adjusted for variables reported to be associated with the likelihood of cesarean section in previous studies, including infant gender, birth weight, maternal age, parity, and education level. The models evaluating maternal pre-pregnancy BMI and BMI/weight gain during pregnancy were mutually adjusted. Analyses were further stratified by maternal pre-pregnancy BMI categories, and we evaluated potential interactions between predictor variables by including relevant cross-product terms in the logistic regression models. Linear trends were tested using the Wald test. Statistical analyses were conducted using SAS, version 9.4, (SAS Institute, Inc., Cary, North Carolina), and a *P* value < 0.05 was considered statistically significant.

## Additional Information

**How to cite this article**: Xiong, C. *et al.* Association of pre-pregnancy body mass index, gestational weight gain with cesarean section in term deliveries of China. *Sci. Rep.* **6**, 37168; doi: 10.1038/srep37168 (2016).

**Publisher’s note:** Springer Nature remains neutral with regard to jurisdictional claims in published maps and institutional affiliations.

## Figures and Tables

**Table 1:** Distribution of selected characteristics among cesarean and vaginal deliveries.

Maternal characteristic	Vaginal deliveries (23,714)		Cesarean deliveries (34,177)	
	n	%	n	%
*Age at delivery (years)*				
<25	5,601	23.62	6,079	17.79
25–29	12,430	52.42	17,483	51.15
30–34	4,565	19.25	8,193	23.97
≥35	1,118	4.71	2,422	7.09
*Education Level*				
Less than high school	3,336	14.07	3,741	10.95
High school	10,317	43.51	16,447	48.12
College	8,655	36.50	12,890	37.72
Advanced degree	1,406	5.93	1,099	3.22
*Offspring Gender*				
Male	12,122	51.12	18,500	54.13
Female	11,592	48.88	15,677	45.87
*Offspring birth weight (g)*				
<2,500	346	1.46	378	1.11
2,500–3,999	22,536	95.03	31,357	91.75
≥4,000	832	3.51	2,442	7.15
*Number of Parity*				
1	18,923	79.80	28,679	83.91
≥2	4,791	20.20	5,498	16.09
*Pre-pregnancy BMI (kg/m*^*2*^)				
Under weight (<18.5)	4,547	19.17	5,574	16.31
Normal (18.5–23.9)	18,251	76.96	26,271	76.87
Overweight (24–27.9)	842	3.55	2,035	5.95
Obese (≥28)	74	0.31	297	0.87

**Table 2:** Associations of pre-pregnancy BMI, GWG, and gestational BMI gain with risk of cesarean section

Variables	Vaginal deliveries (n)	Cesarean deliveries (n)	Crude OR (95% CI)	Adjusted OR (95% CI)
*Pre-pregnancy BMI (kg/m*^*2*^)*				
Under weight (<18.5)	4,547	5,574	0.85(0.82–0.89)	0.83(0.79–0.86)
Normal (18.5–23.9)	18,251	26,271	1.00 (ref)	1.00 (ref)
Overweight (24–27.9)	842	2,035	1.68(1.55–1.82)	1.73(1.59–1.88)
Obese (≥28)	74	297	2.79(2.16–3.60)	2.90(2.24–3.76)
GWG By IOM Recommendation*				
Below	5,086	5,341	0.89(0.84–0.93)	0.90(0.86–0.95)
Within	7,898	9,371	1.00 (ref)	1.00 (ref)
Above	10,730	19,465	1.53(1.47–1.59)	1.45(1.40–1.51)
*Gestational BMI gain (kg/m*^*2*^)*				
<4	4,052	4,300	1.00 (ref)	1.00 (ref)
4–6	6,961	8,176	1.11(1.05–1.17)	1.12(1.06–1.18)
6–8	7,497	10,747	1.35(1.28–1.42)	1.38(1.31–1.46)
≥8	5,204	10,954	1.98(1.88–2.09)	2.01(1.90–2.12)
*P* for trend				<0.01

^*^Adjusted for maternal age at delivery, education level, parity, offspring gender and birth weight. Additionally, pre-pregnancy BMI and gestational BMI gain were mutually adjusted. GWG were also adjusted for pre-pregnancy BMI.

**Table 3:** Associations of GWG and gestational BMI gain with risk of cesarean section stratified by pre-pregnancy BMI.

Variables	Under-weight (<18.5 kg/m^2^)		Normal (18.5–23.9 kg/m^2^)		Overweight/Obese (≥24 kg/m^2^)		*P* for heterogeneity
	Crude OR (95% CI)	Adjusted OR (95% CI)	Crude OR (95% CI)	Adjusted OR (95% CI)	Crude OR (95% CI)	Adjusted OR (95% CI)	
Total GWG By IOM Recommendation*							
Below	0.77(0.68–0.87)	0.78(0.69–0.89)	0.88(0.83–0.92)	0.91(0.86–0.96)	1.17(0.90–1.53)	1.21(0.92–1.58)	0.02
Within	1.00 (ref)	1.00 (ref)	1.00 (ref)	1.00 (ref)	1.00 (ref)	1.00 (ref)	
Above	1.54(1.41–1.67)	1.51(1.38–1.64)	1.48(1.41–1.54)	1.44(1.37–1.50)	1.60(1.33–1.93)	1.56(1.30–1.88)	
*Gestational BMI gain (kg/m*^*2*^) *							
<4	1.00 (ref)	1.00 (ref)	1.00 (ref)	1.00 (ref)	1.00 (ref)	1.00 (ref)	
4–6	1.29(1.07–1.55)	1.25(1.04–1.51)	1.16(1.09–1.23)	1.12(1.05–1.18)	1.12(0.92–1.36)	1.10(0.90–1.33)	<0.01
6–8	1.65(1.38–1.98)	1.60(1.33–1.92)	1.43(1.35–1.52)	1.35(1.28–1.44)	1.53(1.24–1.89)	1.49(1.21–1.85)	
≥8	2.62(2.18–3.16)	2.49(2.07–3.01)	2.10(1.97–2.23)	1.95(1.83–2.07)	2.06(1.58–2.67)	1.92(1.47–2.51)	
*P* for trend		<0.01		<0.01		<0.01	

^*^Adjusted for maternal age at delivery, education level, parity, offspring gender and birth weight.

## References

[b1] Lumbiganon P. *et al.* Method of delivery and pregnancy outcomes in Asia: the WHO global survey on maternal and perinatal health 2007–08. The Lancet 375, 490–499 (2010). 10.1016/S0140-6736(09)61870-5 20071021

[b2] Wispelwey B. P. & Sheiner E. Cesarean delivery in obese women: a comprehensive review. The Journal of Maternal-Fetal & Neonatal Medicine 26, 547–551 (2013). 23130683 10.3109/14767058.2012.745506

[b3] Chu S. Y. *et al.* Association between obesity during pregnancy and increased use of health care. New England Journal of Medicine 358, 1444–1453 (2008). 18385496 10.1056/NEJMoa0706786

[b4] Wu S., Kocherginsky M. & Hibbard J. U. Abnormal placentation: twenty-year analysis. American journal of obstetrics and gynecology 192, 1458–1461 (2005). 15902137 10.1016/j.ajog.2004.12.074

[b5] Li H., Zhou Y. & Liu J. The impact of cesarean section on offspring overweight and obesity: a systematic review and meta-analysis. International journal of obesity 37, 893–899 (2013). 23207407 10.1038/ijo.2012.195

[b6] Dzakpasu S. *et al.* Contribution of prepregnancy body mass index and gestational weight gain to caesarean birth in Canada. BMC pregnancy and childbirth 14, 1 (2014). 24641703 10.1186/1471-2393-14-106 PMC3995143

[b7] Yazdani S., Yosofniyapasha Y., Nasab B. H., Mojaveri M. H. & Bouzari Z. Effect of maternal body mass index on pregnancy outcome and newborn weight. BMC research notes 5, 34 (2012). 22251801 10.1186/1756-0500-5-34 PMC3292487

[b8] Swank M. L. *et al.* The impact of change in pregnancy body mass index on cesarean delivery. The Journal of Maternal-Fetal & Neonatal Medicine 27, 795–800 (2014). 24047475 10.3109/14767058.2013.845657

[b9] Zhou Y. *et al.* Maternal Obesity, Caesarean Delivery and Caesarean Delivery on Maternal Request: a Cohort Analysis from China. Paediatric and perinatal epidemiology 29, 232–240 (2015). 25827169 10.1111/ppe.12191

[b10] O’Dwyer V. *et al.* The risk of caesarean section in obese women analysed by parity. European Journal of Obstetrics & Gynecology and Reproductive Biology 158, 28–32 (2011). 21596472 10.1016/j.ejogrb.2011.04.007

[b11] Koyanagi A. *et al.* Macrosomia in 23 developing countries: an analysis of a multicountry, facility-based, cross-sectional survey. The Lancet 381, 476–483 (2013). 10.1016/S0140-6736(12)61605-5 23290494

[b12] Pallasmaa N., Ekblad U., Gissler M. & Alanen A. The impact of maternal obesity, age, pre-eclampsia and insulin dependent diabetes on severe maternal morbidity by mode of delivery—a register-based cohort study. Archives of gynecology and obstetrics 291, 311–318 (2015). 25115277 10.1007/s00404-014-3352-z

[b13] Poobalan A., Aucott L., Gurung T., Smith W. C. S. & Bhattacharya S. Obesity as an independent risk factor for elective and emergency caesarean delivery in nulliparous women–systematic review and meta‐analysis of cohort studies. Obesity Reviews 10, 28–35 (2009). 19021871 10.1111/j.1467-789X.2008.00537.x

[b14] Chin J. R., Henry E., Holmgren C. M., Varner M. W. & Branch D. W. Maternal obesity and contraction strength in the first stage of labor. American journal of obstetrics and gynecology 207129, e121–e129. e126 (2012). 10.1016/j.ajog.2012.06.044 22840723

[b15] Preiss K., Brennan L. & Clarke D. A systematic review of variables associated with the relationship between obesity and depression. Obesity Reviews 14, 906–918 (2013). 23809142 10.1111/obr.12052

[b16] Bodnar L. M., Wisner K. L., Moses-Kolko E., Sit D. K. & Hanusa B. H. Prepregnancy body mass index, gestational weight gain, and the likelihood of major depressive disorder during pregnancy. The Journal of clinical psychiatry 70, 1290–1296 (2009). 19607761 10.4088/JCP.08m04651 PMC2760651

[b17] Graham L. E., Brunner Huber L. R., Thompson M. E. & Ersek J. L. Does amount of weight gain during pregnancy modify the association between obesity and cesarean section delivery? Birth 41, 93–99 (2014). 24654641 10.1111/birt.12095

[b18] Cho E.-H., Hur J. & Lee K.-J. Early Gestational Weight Gain Rate and Adverse Pregnancy Outcomes in Korean Women. PloS one 10, e0140376 (2015). 26465322 10.1371/journal.pone.0140376 PMC4605500

[b19] Gante I., Amaral N., Dores J. & Almeida M. C. Impact of gestational weight gain on obstetric and neonatal outcomes in obese diabetic women. BMC pregnancy and childbirth 15, 249 (2015). 26449278 10.1186/s12884-015-0692-z PMC4599662

[b20] Bogaerts A., Witters I., Van den Bergh B. R., Jans G. & Devlieger R. Obesity in pregnancy: Altered onset and progression of labour. Midwifery 29, 1303–1313 (2013). 23427851 10.1016/j.midw.2012.12.013

[b21] Bei‐Fan Z. Predictive values of body mass index and waist circumference for risk factors of certain related diseases in Chinese adults: study on optimal cut‐off points of body mass index and waist circumference in Chinese adults. Asia Pacific journal of clinical nutrition 11, S685–S693 (2002). 12046553

[b22] Yaktine A. L. & Rasmussen K. M. Weight Gain During Pregnancy: Reexamining the Guidelines (National Academies Press, 2009). 20669500

[b23] Yang S. *et al.* Parental Body Mass Index, Gestational Weight Gain, and Risk of Macrosomia: a Population‐Based Case–Control Study in China. Paediatric and perinatal epidemiology 29, 462–471 (2015). 26228295 10.1111/ppe.12213

